# Psychiatric disorders after traumatic intracranial hemorrhage: the HEAD Helsinki study

**DOI:** 10.1007/s00701-025-06696-0

**Published:** 2025-10-18

**Authors:** Janne Kinnunen, Jukka Putaala, Ivan Marinkovic, Jarno Satopää, Mika Niemelä, Risto Vataja

**Affiliations:** 1 Department of Neurology, Meilahti Hospital, University of Helsinki and HUS Helsinki University Hospital, Helsinki, Finland; 2 Department of Neurosurgery, Meilahti Hospital, University of Helsinki and HUS Helsinki University Hospital, Helsinki, Finland; 3 Department of Psychiatry, Psychiatry Center, University of Helsinki and HUS Helsinki University Hospital, Helsinki, Finland

**Keywords:** Head trauma, Head injury, Traumatic brain injury, Psychiatric disorders

## Abstract

**Background:**

The extent of psychiatric disorders after head injury is not well recognized. We assessed the prevalence and treatment of psychiatric disorders after traumatic intracranial hemorrhage (tICH) in a 10-year follow-up.

**Methods:**

An observational, retrospective single-center cohort of tICH patients from HEAD Helsinki (Head trauma related health care Economics, Acute care and Development of long-term outcomes in Helsinki city region) study hospitalized at Helsinki University Hospital between 01 January and 31 December 2010. We reported primary outcomes as psychiatric disorders and their subsequent treatment on secondary level psychiatric care during the follow-up period between January 2010 and December 2019. Logistic regression analysis was performed to study associations between admission clinical characteristics and primary outcomes.

**Results:**

In our cohort of 385 patients (mean age 60.7 years, 66.5% male) with tICH, 66 (17.1%) had any psychiatric disorders during the follow-up period (median time 108 months, IQR 92 months), and 48 (72.1%) of them experienced new psychiatric disorders (median time to onset 29.5 months, IQR 64 months), of which 26 (54.2%) were without any psychiatric history prior to tICH. A total of 35 patients (53.0%) received secondary level psychiatric care, and 40 (60.6%) patients had initiated new psychotropics (median time to initiation 8 months, IQR 40 months). Compared to patients without psychiatric disorders, those with psychiatric disorders were younger (mean age 49.1 vs. 63.1 years, *p* < 0.001) and had less frequently larger (> 100 ml) intracranial hemorrhages (21.2 vs. 44.2%, *p* = 0.001). In multivariable analyses, younger age was independently associated with the development of any psychiatric disorders. Prior psychiatric medication and lower admission GCS score were associated with consequent psychiatric treatment.

**Conclusions:**

Every sixth patient treated in a tertiary level neurosurgical unit had psychiatric disorders diagnosed at secondary level care after tICH and over half of them received secondary level psychiatric care. Psychiatric disorders development was associated with younger age whereas prior psychiatric medication and lower admission GCS score indicated subsequent psychiatric treatment.

## Introduction

Traumatic brain injury (TBI) has a potential of severe long-term consequences [20]. Worldwide, TBI causes mortality and disability more than any other traumatic event, and within high-income countries it is the leading cause of disability among the young due to its increasing incidence [5, 14]. In 2016, there were approximately 27 million new cases of TBIs, with a prevalence of 55 million [9]. The basic classification of TBI is a division between primary brain injury due to direct traumatic force effect to the cranium and secondary brain injury occurring later after the initial episode [1]. TBIs are also divided into subtypes depending on the injury component: diffuse axonal injury (DAI) contains microhemorrhages, whereas contusions, epidural hemorrhages (EDH) and subdural hemorrhages (SDH), and subarachnoid hemorrhages (SAH) represent more vast damages regarding the hemorrhagic component itself [24] which are defined as traumatic intracranial hemorrhages (tICH) in this study.

Psychiatric disorders following TBI are a common manifestation but remain understudied and only partially understood [2], also not included in the traditional TBI classification system [18]. It has been reported that almost 40% of patients suffer some degree of psychiatric disorders after TBI [30].

Studies have shown that patients with more severe TBIs are more likely to develop more severe psychiatric disorders, with the majority experiencing cognitive disorders (92%), personality changes (88%) and mood disorders (67%) [13]. The severity of disorders influences the level and type of psychiatric care provided [7].

The need for early identification and evaluation of psychiatric symptoms in patients with more severe TBI has been recognized [31]. Yet, the spectrum and treatment of psychiatric disorders in this patient group remains underrecognized.

Our aim was to study the prevalence of psychiatric disorders diagnosed at secondary level care after tICH over a 10-year follow-up period, and the proportion of patients receiving treatment in secondary level psychiatric care, and baseline clinical factors associated with psychiatric morbidity.

## Methods

Our study was conducted as an observational, retrospective single-center cohort of consecutive adult (aged 18 years or older) tICH patients from HEAD Helsinki (Head trauma related health care Economics, Acute care and Development of long-term outcomes in Helsinki city region) study hospitalized at Helsinki University Hospital between 01 January and 31 December 2010 [10]. Our hospital is serving 24/7 as the only neurosurgical and neurocritical emergency unit for catchment population of 2.2 million. The study was approved by HUS (Helsinki and Uusimaa Hospital District) and institutional permission was obtained (HUS/216/2023). Patient consent was waived as the study was based on data collected during clinical evaluation and treatment, without additional patient contact.

Initially, we screened the tICH patients from all patients being treated in our hospital emergency with a suspected TBI according to hospital discharge registry, using International Classification of Diseases, 10th version (ICD-10) codes S06.*, I60.*, I61.* and I62.*. Subsequently, we excluded patients with no indication of TBI, non-traumatic intracranial hemorrhage, or inadequate clinical or radiological data. Of these, we included patients residing in the Helsinki and Uusimaa Hospital District with complete follow-up data available.

Data was collected from electronic patient charts of secondary hospital register for clinical characteristics and radiological imaging. Alcohol use was estimated based on patients records and laboratory findings indicative for abusive consumption. In Finland, the source of data for pathological alcohol usage is based on reliable register data on alcohol-related visit in hospital care, and this data source has been validated [16]. Accordingly, our data on abusive alcohol usage is based on trustworthy documented hospital visits, and these were evaluated manually by a researcher (JK). We defined one standard alcohol drink as 12 g of pure alcohol, and for heavy drinking > 20 g/day or 12 to 16 drinks/week for women and > 40 g/day or 23 to 24 drinks/week for men [8, 26].

On hospital admission, patients were evaluated for state of consciousness with Glasgow Coma Scale (GCS) score, and they underwent radiological imaging of head by computerized tomography (CT). CT scans were evaluated by a staff on-call radiologist or neuroradiologist stating any presence of tICH. Possible surgical treatment was evaluated by on-call neurosurgeon. Any neurosurgical modality for evacuation of hemorrhage was considered as neurosurgical intervention. In retrospective, a researcher (JK) collected and evaluated all related CT images to confirm TBI findings. If there were multiple intracranial hemorrhage components, the main one was chosen for the analysis, and its volume was measured by ABC/2 and XYZ/2 methods [12, 28].

Patient follow-up was conducted using secondary hospital register by reviewing patient charts between the years 2010 and 2019. End points were new psychiatric disorder diagnosed at secondary level care (ICD codes), patient’s death or end of study follow-up period. Psychiatric disorders were categorized based on previous literature comprising all the relevant symptom groups in post-TBI evaluation: depression, anxiety disorders, bipolar disorder, suicidal behavior, psychotic episodes, delirium, personality disorders, post-traumatic stress disorder (PTSD) and psychiatric disorders due to intoxicating psychoactive substance use. We collected data on previous diagnoses of any psychiatric disorders and psychotropic medication listed on patient charts. Also, psychiatric disorders and related medication use after tICH was evaluated based on patient records’ information of diagnoses and psychotropics initiation interpreted by the same researcher (JK).

Statistical analyses were performed by SPSS 29.0 (IBM Corp., Armonk, NY, USA). Normality of distributions was assessed. Student’s t test was used for normally distributed continuous variables and Mann–Whitney U test for non-normally distributed continuous variables. Pearson’s chi-square and Fisher’s exact test allowed comparison of the categorical variables. Multivariable logistic regression was used to assess factors associated with psychiatric disorders and follow-up secondary level psychiatric treatment. A two-sided *p* < 0.05 was considered significant. Covariable selection was done in referral to existing literature and justification of clinical aspects related to the incident head injury event, including demographics (age, sex), patient history (alcohol abuse, stroke, traumatic brain injuries, psychiatric morbidity and medication), clinical features (cognitive problems on admission, GCS, hemorrhage volumes, neurosurgical intervention), type of tICH, duration of hospital stay and place of discharge.

## Results

**Fig. 1:**
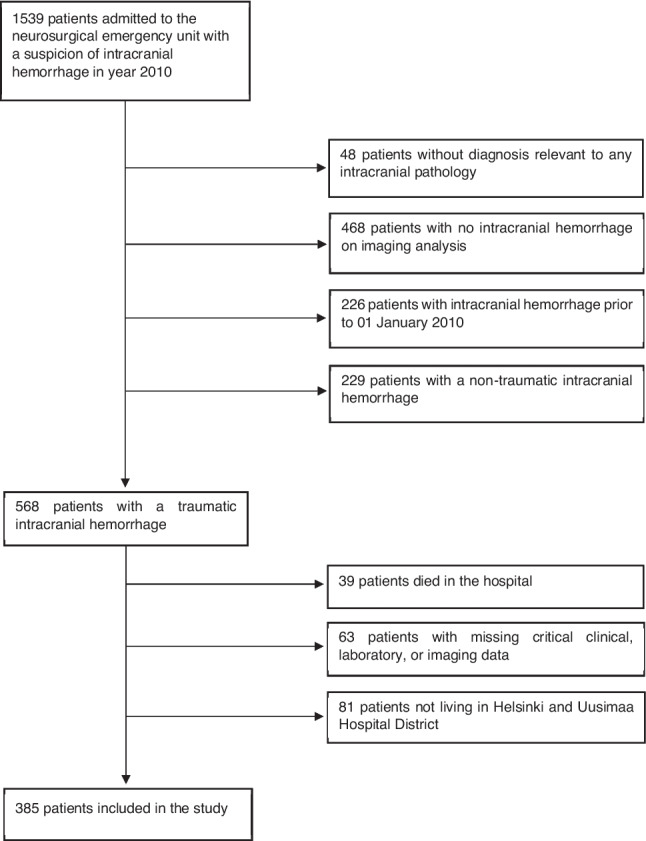
Patient selection flowchart

**Fig. 2:**
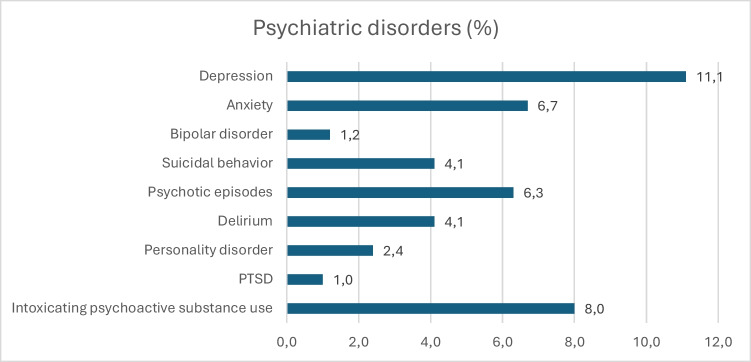
Prevalence of psychiatric disorders during the follow-up in the entire study cohort

Altogether 1539 patients were admitted to our neurosurgical emergency unit in 2010 with a suspicion of TBI. Of these, 505 patients were imaging positive for tICH, and of them 385 were resided within the confines of Helsinki and Uusimaa Hospital District and were included in the follow-up (Fig. 1). Among them, the prevalences of psychiatric disorders were as follows: depression 11.1%, anxiety 6.7%, bipolar disorder 1.2%, suicidal behavior 4.1%, psychotic episodes 6.3%, delirium 4.1%, personality disorders 2.4%, PTSD 1.0%, intoxicating psychoactive substance use 8.0% (Fig. 2). The median follow-up time was 108 months (interquartile range (IQR) 92 months). After tICH, the median time to onset of a new psychiatric disorder was 29.5 months (IQR 64 months) and initiation of a new psychotropic medication 8 months (IQR 40 months).

### Patients with any psychiatric disorders during the follow-up

**Fig. 3:**
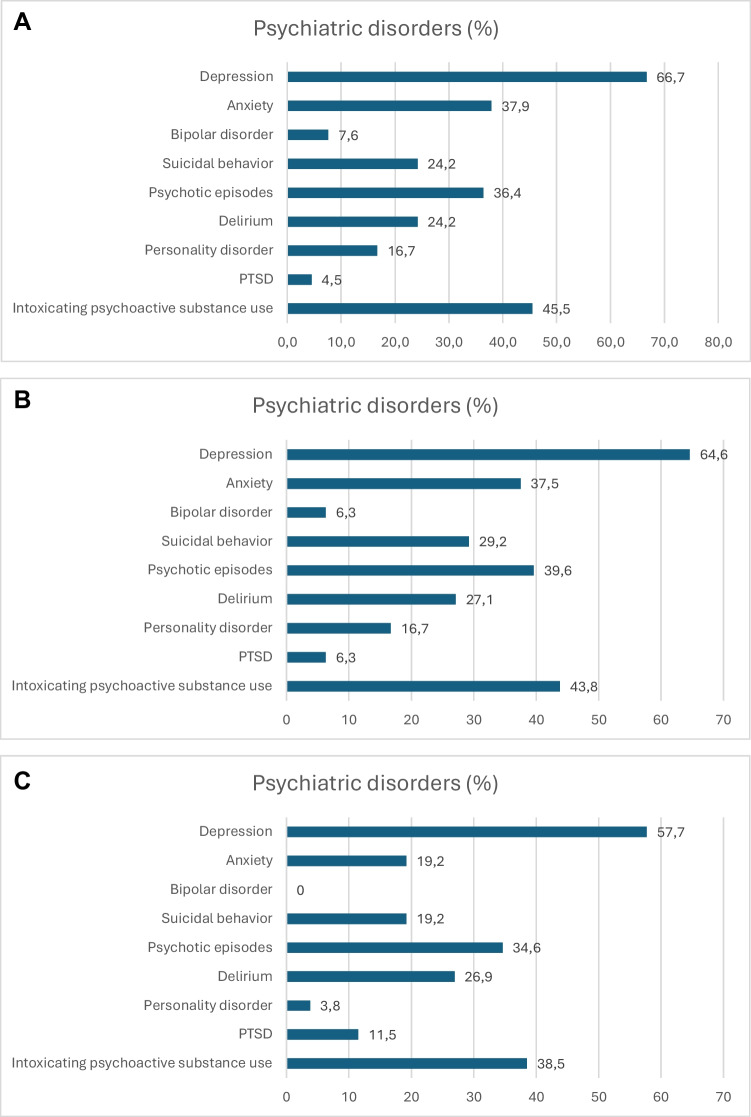
**A** Distribution of psychiatric disorders during the follow-up in patients with any psychiatric disorders. **B** Distribution of psychiatric disorders during the follow-up in patients with newly emerged psychiatric disorders. **C** Distribution of psychiatric disorders during the follow-up in patients without previous psychiatric morbidity

Of the included patients, 256 (66.5%) were male, and the mean age was 60.7 years. Psychiatric disorders occurred in 66 (17.1%) patients (presenting with one or more psychiatric disorders), who were mostly male (65.2%), and the mean age was 49.1 years (Table 1). The three most common psychiatric disorders in this patient group were depression, intoxicating psychoactive substance use, and anxiety (Fig. 3A). Of these, 35 patients (53.0%) were receiving secondary level psychiatric treatment during the follow-up.

**Table 1:** Any psychiatric disorders after traumatic intracranial hemorrhage. Univariable and multivariable analysis of associated factors. Odds ratios from a logistic regression model: analyzing each variable separately and adjusted for all the given variables

	Psychiatric disorders *N* = 66 (17.1%)	No psychiatric disorder *N* = 319 (82.9%)	Univariable OR (95% CI)	Univariable* p*	Multivariable OR (95% CI)	Multivariable *p*
Sex, male, *n* (%)	43 (65.2)	213 (66.8)	0.948 (0.544–1.652)	0.850	0.743 (0.397–1.392)	0.354
Age, mean (95% CI)	49.1 (44.5–53.7)	63.1 (61.2–65.0)	0.967 (0.953–0.980)	< 0.001	NA^a^	
Age group						
≤ 50	34 (51.5)	61 (19.1)	6.689 (3.307–13.527)	< 0.001	7.039 (3.120–15.882)	< 0.001
51–64	19 (28.8)	102 (32.0)	2.235 (1.058–4.724)	0.035	2.038 (0.902–4.605)	0.087
≥ 65	13 (19.7)	156 (48.9)	Reference		Reference	
Alcohol abuse	24 (36.4)	93 (29.2)	1.438 (0.824–2.507)	0.201	1.213 (0.642–2.290)	0.552
Previous stroke, n (%)	5 (7.6)	31 (9.7)	0.783 (0.293–2.094)	0.625	NA^b^	
Previous tICH, n (%)	3 (4.5)	19 (6.0)	0.772 (0.222–2.687)	0.684	NA^b^	
Admission GCS						
13–15	45 (68.2)	220 (69.0)	Reference		Reference	
9–12	6 (9.1)	37 (11.6)	0.804 (0.320–2.017)	0.641	0.655 (0.234–1.835)	0.420
3–8	15 (22.7)	62 (19.4)	1.109 (0.582–2.115)	0.752	0.957 (0.390–2.346)	0.924
Admission cognition problem	36 (54.5)	152 (47.6)	1.348 (0.793–2.292)	0.270	1.342 (0.745–2.419)	0.327
Type of tICH						
Contusion	11 (16.7)	46 (14.4)	Reference		Reference	
EDH/SDH/SAH	55 (83.3)	273 (85.6)	0.842 (0.411–1.729)	0.640	1.802 (0.785–4.134)	0.165
Hemorrhage volume (ml)						
0–50	40 (60.6)	140 (43.9)	Reference		Reference	
51–100	12 (18.2)	38 (11.9)	0.988 (0.464–2.105)	0.976	1.262 (0.509–3.128)	0.616
> 100	14 (21.2)	141 (44.2)	0.339 (0.177–0.650)	0.001	0.425 (0.165–1.096)	0.077
Neurosurgical operation	29 (43.9)	180 (56.4)	0.617 (0.362–1.051)	0.075	1.191 (0.536–2.650)	0.668
Hospital stay in days, median (IQR)	7 (9)	6 (9)	1.005 (0.978–1.034)	0.702	1.006 (0.960–1.055)	0.791
ICU stay in days, median (IQR)	1 (4)	0 (4)	1.008 (0.955–1.064)	0.776	0.957 (0.882–1.038)	0.284
Discharge destination			N/A		N/A	
Home	20 (30.3)	105 (32.9)				
Hospital or nursery	46 (69.7)	214 (67.1)				

*OR* odds ratio, *CI* confidence interval, *p* *p*-value, *IQR* interquartile range, *GCS* Glasgow Coma Scale, *tICH* traumatic intracranial hemorrhage, *EDH* epidural hemorrhage, *SDH* subdural hemorrhage, *SAH* subarachnoidal hemorrhage, *ICU* intensive care unit, *NA*^a^ = not included in the regression model due to categorized parameter of the same value, *NA*^b^ = not reported due to lack of adequate number of observations, N/A not applicable since reported only in quantity

Compared to those without any psychiatric morbidity, patients with psychiatric disorders were younger (mean age 49.1 vs 63.1 years), had less neurosurgical operations (43.9 vs 56.4%), and developed smaller intracranial hemorrhage volumes (21.2 vs 44.2%). Incidences of pooled extra-axial tICH subtypes were, respectively: EDH 9.1 vs 2.5%, SDH 74.2 vs 78.1%, and SAH 0.0 vs 5.0%. In multivariable analysis, younger age was associated with psychiatric disorders after tICH (Table 1).

### Patients with newly emerged psychiatric disorders during the follow-up

New psychiatric disorders, regardless of previous psychiatric morbidity, emerged in 48 (12.5%) patients, the majority of whom were men (67.2%) with mean age of 47.5 years (Table 2). The three most common psychiatric disorders in this patient group were depression, intoxicating psychoactive substance use, and psychotic episodes (Fig. 3B). Of these, 28 patients (58.3%) were receiving secondary level psychiatric treatment during the follow-up period.

**Table 2:** Newly emerged psychiatric disorders after traumatic intracranial hemorrhage. Univariable and multivariable analysis of associated factors. Odds ratios from a logistic regression model: analyzing each variable separately and adjusted for all the given variables

	New psychiatric disorders *N* = 48 (12.5%)	No new psychiatric disorders *N* = 337 (87.5%)	Univariable OR (95% CI)	Univariable *p*	Multivariable OR (95% CI)	Multivariable *p*
Sex, male, n (%)	39 (67.2)	224 (66.5)	1.009 (0.531–1.916)	0.978	0.833 (0.403–1.723)	0.623
Age, mean (95% CI)	47.5 (42.6–52.3)	62.7 (60.9–64.6)	0.954 (0.938–0.971)	< 0.001	NA^a^	
Age group						
≤ 50	28 (58.3)	67 (19.9)	9.672 (4.029–23.220)	< 0.001	8.672 (3.248–23.157)	< 0.001
51–64	13 (27.1)	108 (32.0)	2.786 (1.077–7.207)	0.035	2.392 (0.857–6.674)	0.096
≥ 65	7 (14.6)	162 (48.1)	Reference		Reference	
Alcohol abuse	17 (35.4)	100 (29.7)	1.014 (0.984–1.044)	0.370	1.061 (0.514–2.187)	0.874
Previous stroke, n (%)	2 (4.2)	34 (10.1)	0.387 (0.090–1.667)	0.203	NA^b^	
Previous tICH, n (%)	1 (2.1)	21 (6.2)	0.320 (0.042–2.436)	0.271	NA^b^	
Admission GCS						
13–15	30 (62.5)	235 (69.7)	Reference		Reference	
9–12	6 (12.5)	37 (11.0)	1.270 (0.495–3.260)	0.619	1.087 (0.372–3.178)	0.879
3–8	12 (25.0)	65 (19.3)	1.446 (0.701–2.982)	0.318	1.131 (0.415–3.084)	0.810
Admission cognition problem	28 (58.3)	160 (47.5)	1.549 (0.840–2.857)	0.161	1.503 (0.765–2.954)	0.237
Type of tICH						
Contusion	10 (20.8)	47 (13.9)	Reference		Reference	
EDH/SDH/SAH	38 (79.2)	290 (86.1)	0.616 (0.288–1.319)	0.212	1.411 (0.587–3.390)	0.441
Hemorrhage volume (ml)						
0–50	33 (68.8)	147 (43.6)	Reference		Reference	
51–100	6 (12.5)	44 (13.1)	0.626 (0.246–1.592)	0.325	0.951 (0.319–2.836)	0.928
> 100	9 (18.7)	146 (43.3)	0.276 (0.128–0.598)	0.001	0.480 (0.161–1.434)	0.189
Neurosurgical operation	19 (39.6)	190 (56.4)	0.507 (0.273–0.940)	0.031	0.959 (0.385–2.391)	0.929
Hospital stay in days, median (IQR)	10 (10)	6 (8)	1.014 (0.984–1.044)	0.370	1.015 (0.965–1.066)	0.567
ICU stay in days, median (IQR)	2 (5)	0 (4)	1.024 (0.966–1.085)	0.423	0.962 (0.881–1.051)	0.394
Discharge destination			N/A		N/A	
Home	16 (33.3)	109 (32.3)				
Hospital or nursery	32 (66.7)	228 (67.7)				

*OR* odds ratio, *CI* confidence interval, *p* *p*-value, *IQR* interquartile range, *GCS* Glasgow Coma Scale, *tICH* traumatic intracranial hemorrhage, *EDH* epidural hemorrhage, *SDH* subdural hemorrhage, *SAH* subarachnoidal hemorrhage, *ICU* intensive care unit, *NA*^a^  not included in the regression model due to categorized parameter of the same value, *NA*^b^ not reported due to lack of adequate number of observations, *N/A* not applicable since reported only in quantity

Patients with new psychiatric disorders were younger in comparison to those without any psychiatric disorders (mean age 47.5 vs 62.7 years), had more cognition problems on admission (58.3 vs 47.5%), and developed smaller intracranial hemorrhage volumes (18.7 vs 43.3%). Incidences of pooled extra-axial tICH subtypes were, respectively: EDH 10.4 vs 2.7%, SDH 68.8 vs 78.6%, and SAH 0.0 vs 4.7%. In multivariable analysis, younger age was associated with new psychiatric disorders after tICH (Table 2).

### Patients with psychiatric disorders during the follow-up without previous psychiatric morbidity

Altogether 26 patients (8.0%) had new psychiatric disorders without any previous psychiatric history, of them majority were male (69.2%), and their mean age was 46.5 years (Table 3). The most common psychiatric disorders in this patient group were depression, intoxicating psychoactive substance use, and psychotic episodes (Fig. 3C). Of these, 10 patients (38.5%) received secondary level psychiatric treatment during the follow-up period.

**Table 3:** Psychiatric disorders after traumatic intracranial hemorrhage in patients without previous psychiatric morbidity. Univariable and multivariable analysis of associated factors. Odds ratios from a logistic regression model: analyzing each variable separately and adjusted for all the given variables

	Psychiatric disorders w/o psychiatric history *N* = 26 (8.0%)	No psychiatric disorders w/o psychiatric history *N* = 299 (92.0%)	Univariable OR (95% CI)	Univariable *p*	Multivariable OR (95% CI)	Multivariable *p*
Sex, male, *n* (%)	18 (69.2)	200 (66.9)	1.114 (0.468–2.650)	0.808	0.830 (0.310–2.221)	0.711
Age, mean (95% CI)	46.5 (40.1–53.0)	63.1 (61.1–65.1)	0.954 (0.933–0.975)	< 0.001	NA^a^	
Age group						
≤ 50	14 (53.8)	56 (18.7)	12.333 (3.414–44.550)	12.333	9.774 (2.453–38.937)	0.001
51–64	9 (34.6)	95 (31.8)	4.674 (1.234–17.703)	0.023	3.199 (0.770–13.292)	0.110
≥ 65	3 (11.5)	148 (49.5)	Reference		Reference	
Alcohol abuse	10 (38.5)	83 (27.8)	1.627 (0.709–3.729)	0.250	1.387 (0.531–3.621)	0.505
Previous stroke, n (%)	1 (3.8)	25 (8.4)	0.438 (0.057–3.373)	0.428	NA^b^	
Previous tICH, n (%)	0 (0.0)	14 (4.7)	NA^b^		NA^b^	
Admission GCS						
13–15	15 (57.7)	207 (69.2)	Reference		Reference	
9–12	4 (15.4)	34 (11.4)	1.624 (0.508–5.184)	0.413	1.346 (0.361–5.020)	0.658
3–8	7 (26.9)	58 (19.4)	1.666 (0.649–4.277)	0.289	1.554 (0.430–5.620)	0.502
Admission cognition problem	15 (57.7)	143 (47.8)	1.488 (0.661–3.345)	0.337	1.256 (0.509–3.096)	0.621
Type of tICH						
Contusion	4 (15.4)	45 (15.1)	Reference		Reference	
EDH/SDH/SAH	22 (84.6)	254 (84.9)	0.974 (0.321–2.961)	0.964	2.450 (0.717–8.377)	0.153
Hemorrhage volume (ml)						
0–50	18 (69.2)	133 (44.5)	Reference		Reference	
51–100	5 (19.2)	36 (12.0)	1.026 (0.357–2.953)	0.962	1.109 (0.319–3.857)	0.870
> 100	3 (11.5)	130 (43.5)	0.171 (0.049–0.593)	0.005	0.289 (0.058–1.441)	0.130
Neurosurgical operation	9 (34.6)	167 (55.9)	0.418 (0.181–0.969)	0.042	0.902 (0.286–2.844)	0.861
Hospital stay in days, median (IQR)	10 (9)	6 (9)	1.005 (0.964–1.048)	0.815	0.993 (0.922–1.070)	0.860
ICU stay in days, median (IQR)	2 (7)	0 (4)	1.026 (0.953–1.105)	0.497	0.962 (0.860–1.075)	0.490
Discharge destination			N/A		N/A	
Home	9 (34.6)	100 (33.4)				
Hospital or nursery	17 (65.4)	199 (66.6)				

*OR* odds ratio, *CI* confidence interval, *p* *p*-value, *IQR* interquartile range, *GCS* Glasgow Coma Scale, *tICH* traumatic intracranial hemorrhage, *EDH* epidural hemorrhage, *SDH* subdural hemorrhage, *SAH* subarachnoidal hemorrhage, *ICU* intensive care unit, *NA*^a^  not included in the regression model due to categorized parameter of the same value, *NA*^b^ not reported due to lack of adequate number of observations, *N/A* not applicable since reported only in quantity

Patients with new psychiatric disorders without previous psychiatric morbidity in comparison to those without any psychiatric disorders were younger (mean age 46.5 vs 63.1 years), had more often alcohol abuse (38.5 vs 27.8%), and their hospital and ICU stay were longer (median 10 vs 6 days and 2 vs 0 days, respectively). Incidences of pooled extra-axial tICH subtypes were, respectively: EDH 15.4 vs 2.7%, SDH 69.2 vs 76.9%, and SAH 0.0 vs 5.4%. In multivariable analysis, younger age was associated with higher likelihood of first-time psychiatric disorders after tICH (Table 3).

### Psychiatric treatment during the follow-up

At least secondary level psychiatric care was initiated in 35 (53.0%) of patients with psychiatric disorders (mean age 43.9 years, 65.7% male) with 25 (71.4%) of them having history of psychiatric morbidity. Therefore, approximately one-third of the patients receiving psychiatric treatment experienced psychiatric disorders without any previous psychiatric morbidity. Incidences of pooled extra-axial tICH subtypes, in comparison between patients receiving psychiatric treatment and those without, were respectively: EDH 14.3 vs 3.2%, SDH 62.9 vs 87.1%, and SAH 0.0 vs 0.0%. In multivariable analysis prior psychiatric medication and lower GCS score were independently associated with psychiatric treatment in secondary level psychiatric care during follow-up, whereas lower tICH volume indicated less need for psychiatric treatment (Table 4).

**Table 4:** Psychiatric treatment after traumatic intracranial hemorrhage. Univariable and multivariable analysis of associated factors. Odds ratios from a logistic regression model: analyzing each variable separately and adjusted for all the given variables

	Psychiatric treatment *N* = 35 (53.0%)	No psychiatric treatment *N* = 31 (47.0%)	Univariable OR (95% CI)	Univariable *p*	Multivariable OR (95% CI)	Multivariable *p*
Sex, male, n (%)	23 (65.7)	20 (64.5)	1.054 (0.382–2.906)	0.919	NA^c^	
Age, mean (95% CI)	43.9 (37.5–50.3)	55.0 (48.8–61.2)	0.966 (0.938–0.994)	0.018	0.976 (0.940–1.012)	0.189
Age group					NA^a^	
≤ 50	24 (68.6)	10 (32.3)	11.087 (4.067–30.227)	< 0.001		
51–64	6 (17.1)	13 (41.9)	1.711 (0.510–5.742)	0.384		
≥ 65	5 (14.3)	8 (25.8)	Reference			
Alcohol abuse	13 (37.1)	11 (35.5)	1.074 (0.393–2.937)	0.889	NA^c^	
Prior psychiatric disorders	25 (71.4)	15 (48.4)	2.667 (0.965–7.372)	0.059	NA^c^	
Prior psychiatric medication	22 (62.9)	10 (32.3)	3.554 (1.284–9.840)	0.001	10.571 (2.507–44.572)	0.001
New psychiatric disorders	28 (28.0)	20 (64.5)	2.200 (0.727–6.661)	0.163	NA^c^	
Admission GCS						
13–15	20 (57.1)	25 (80.6)	Reference		Reference	
9–12	3 (8.6)	3 (9.7)	1.250 (0.227–6.876)	0.798	1.349 (0.164–11.091)	0.781
3–8	12 (34.3)	3 (9.7)	5.000 (1.239–20.177)	0.024	10.307 (1.596–66.579)	0.014
Admission cognition problem	20 (57.1)	16 (51.6)	1.250 (0.473–3.303)	0.653	NA^c^	
Previous stroke, n (%)	2 (5.7)	3 (9.7)	0.566 (0.088–3.629)	0.548	NA^c^	
Previous tICH, n (%)	2 (5.7)	1 (3.2)	1.818 (0.157–21.088)	0.633	NA^c^	
Type of tICH					NA^c^	
Contusion	8 (22.9)	3 (9.7)	Reference			
EDH/SDH/SAH	27 (77.1)	28 (90.3)	0.549 (0.236–1.279)	0.165		
Hemorrhage volume (ml)						
0–50	26 (74.3)	14 (45.2)	Reference		Reference	
51–100	2 (5.7)	10 (32.3)	0.254 (0.058–1.109)	0.068	0.133 (0.020–0.889)	0.037
> 100	7 (20.0)	7 (22.6)	0.282 (0.119–0.669)	0.004	0.566 (0.112–2.858)	0.491
Neurosurgical operation	15 (42.9)	14 (45.2)	0.911 (0.344–2.412)	0.851	NA^c^	
Hospital stay in days, median (IQR)	8 (11)	6 (10)	1.022 (0.965–1.083)	0.453	NA^c^	
ICU stay in days, median (IQR)	2 (5)	0 (2)	1.122 (0.987–1.276)	0.078	NA^c^	
Discharge destination			N/A		N/A	
Home	9 (25.7)	11 (35.5)				
Hospital or nursery	26 (74.3)	20 (64.5)				

*OR* odds ratio, *CI* confidence interval, *p* *p*-value, *IQR* interquartile range, *GCS* Glasgow Coma Scale, *tICH* traumatic intracranial hemorrhage, *EDH* epidural hemorrhage, *SDH* subdural hemorrhage, *SAH* subarachnoidal hemorrhage, *ICU*  intensive care unit, *NA*^a^ not included in the regression model due to categorized parameter of the same value, *NA*^c^ not included in the multivariable regression model due to lack of significance in univariable analysis, *N/A* not applicable since reported only in quantity

## Discussion

Our study demonstrates that a significant portion of tICH patients experienced psychiatric disorders diagnosed at secondary level care after their index tICH. Most of these patients developed new psychiatric disorders and over half of them were experiencing first-time ever psychiatric disorders during the follow-up. Secondary level psychiatric care was needed in over half of these patients referring to more complex pathology.

### Clinical features of the tICH patients having psychiatric disorders

Our study showed younger age being strongly associated with psychiatric morbidity regardless of prior psychiatric history. Older patients, however, may be more susceptible to developing de novo psychiatric disorders following TBI – a pattern that has been previously suggested in the literature [32]. In older patients, the psychiatric diagnostics associated with tICH is often complicated by age-related neurodegenerative psychiatric symptoms. In our study, we did not differentiate between neurological and psychiatric etiology regarding previous psychiatric disorders. Nevertheless, over two-thirds of patients with psychiatric history, as well as those who developed psychiatric disorders after tICH were under the age of 65.

Patients with psychiatric disorders after tICH had less frequently larger intracranial hemorrhage. Although, patients with smaller tICH volumes needed less often psychiatric treatment after trauma. The association between the initial hemorrhage volume of tICH and psychiatric disorders or need for psychiatric treatment has not been widely studied [4]. It can be speculated that patients with greater volume of intracranial hemorrhage have more anosognosia (lack of insight into their psychiatric symptoms), or their rehabilitation may focus more on physical symptoms than psychiatric disorders, leading to fewer psychiatric care contacts [27, 29]. Inversely, patients with smaller hemorrhages have better survival with less mortality [10], which might emphasize the prevalence of psychiatric disorders among them.

Our study did not differentiate between neurosurgical operation modalities and found no association between neurosurgical intervention and psychiatric disorders. Studies of association between neurosurgical procedures and psychiatric disorders in TBI patients are scarce, when compared i.e. with numerous studies of psychiatric complications after epilepsy surgery [22].

### Type of psychiatric disorders in TBI

Epidemiology of post-TBI psychiatric symptoms has been previously studied, suggesting a wide range of prevalence between 18.3–83.3% for any type of psychiatric symptoms [21]. In more specific approach regarding the severity of TBI, the prevalence for any type of psychiatric sequelae is stated 27.9–34.0% for mild TBIs and 26.3–49.1% for moderate to severe TBIs [6]. Our study shows the overall percentage to be lower, approximately 17%, yet our study is focused on secondary level psychiatry which could explain the lesser number of patients with psychiatric disorders. When considering the severity and type of tICH, we did not find any eminent difference in their proportional occurrence in relation to the psychiatric sequelae categories.

In our study, the prevalences of distinct psychiatric disorders represent mainly the lower end of, or less than, previously reported prevalences. For depression, anxiety disorders, and bipolar disorder the prevalences were lower, and for PTSD significantly less than in previous literature [21]. Regarding intoxicating psychoactive substance use, our study reports approximately half of the prevalence of what has been described elsewhere in patients with psychiatric morbidity [21].

Our study reports quite a moderate percentage for suicidal behavior, which was in accordance with another study from Finland [15], while elsewhere it has been previously estimated to be present even with one-fifth of post-TBI patients suffering from mental health morbidity [19].

Regarding the other form of severe psychiatric disorders, psychotic episodes, our study found the prevalence to be within the range of what has been reported in previous studies [25]. Regarding delirium-type disorders, our study finds the prevalence to be quite low in comparison to previous studies which most are centered on acute phase [3, 23].

Personality disorders, requiring more extensive diagnostic procedures and longer follow-up, could remain partially unrecognized due to the focus on more acute psychiatric manifestations. Our study found notably lesser prevalence than a previous Finnish study reported [11].

In general, our study focused on secondary level psychiatry, therefore showing lower levels of prevalence. Further, studies reporting specifically this level of psychiatry treatment post-TBI are scant.

### Follow-up treatment in secondary level psychiatry

Prior psychotropic medication was associated with subsequent psychiatric treatment after tICH. A study reported common psychotropic medication usage among patients with TBI and relevance to its severity including aspect of GCS level [31]. In mild TBIs, one study showed no significancy of GCS level on psychiatric disorders [17]. Our study did not find any association between the level of admission GCS score and prevalent psychiatric disorders, yet there was an independent association between lower GCS score and subsequent secondary level psychiatric treatment indicating that more severe tICH leads to more extensive psychiatric morbidity.

Studies reporting specifically secondary level psychiatric care are limited, one study reports the incidence between 11.7% and 29% [6]. Our study found this level of care being established in 45.8% of patients having post-TBI psychiatric disorders. This difference might be methodologically explained, where our study consists of younger population having more of the psychiatric disorders post-TBI as indicated in our study. Also, our catchment for secondary level psychiatric care patients involves many types of treatment contacts, not only hospitalizations in psychiatric ward, but visits in other psychiatric facilities such as secondary level acute or specialized psychiatric outpatient clinics.

### Strengths and limitations

The retrospective nature and single-center cohort are the main limitations of our study. However, this type of set up has also strengths as we were able to gather consecutive patients from a well-defined and significant sized metropolitan catchment area and the study is free of consent bias. Many tICH patients may have possessed different comorbidities or intoxication status, which may interfere obtainment of written consent. Our cohort concentrates on more severe head traumas because these patients were admitted to a secondary trauma hospital specializing in tertiary level neurosurgical care leaving out most of the mild TBIs and those not admitted to the hospital, which biases selection of patients toward more severe head injuries and limits generalizability. Our exclusion criteria did not allow analyzing milder TBIs further. Also, we chose not to differentiate between background diseases which might possess neuropsychological propensities to develop further psychiatric sequelae, for example neurodegenerative diseases. We also did not segregate between pre-existing psychiatric disorders, thereof emphasizing equally any previous psychiatric morbidity. Our cohort represents, not the total prevalence of psychiatric morbidity after tICH, but those ended up to secondary level psychiatric evaluation.

## Conclusions

The extent of psychiatric disorders diagnosed at secondary level care after tICH treated in a tertiary level neurosurgical unit appears significant and their severity is prominent. Most of the patients with psychiatric disorders were experiencing them for the first time in their life after a tICH. Younger age was an indicator for development of psychiatric disorders after tICH. History of psychiatric medication and lower admission GCS score were associated with psychiatric care at secondary level which was needed in notable number of tICH survivors.

## Data Availability

Data sharing is not available due to privacy and security concerns.
